# Our Bureau of Information

**Published:** 1911-12-02

**Authors:** 


### OUR BUREAU OF INFORMATION.

### Will Our Readers Please Lend a Hand.

Every week it becomes more clear that the Bureau needs the co-operation of a great number of correspondents up and down the United Kingdom, who are interested in institutional work or engaged as district visitors, secretaries of institutions, teachers, students, healthy young people of both sexes of mature years who have leisure, and all who desire to live and not merely to exist. The work is a good work, which must prove of practical utility if we can awaken the interest and secure the co-operation needed to make it as widely effective and useful as possible. Our letter-box proves, as we knew at the commencement, that there are scattered about the country a number of sufferers, either physically ill or lacking some equipment for life's battle, the want of which can be supplemented by knowledge and kindly counsel, or the application of some means already in existence which experience can bring to bear in individual cases. What a different country ours would be if every family contained at least one friendly worker whose mission it was to be neighbourly and helpful to those in their vicinity less fortunately circumstanced than themselves.

### What We are After.

But, apart from this, homes and the cost of maintaining families are often made infinitely difficult for the want of the co-operative advice and help which an organisation like that we are endeavouring to make a reality, through the Bureau, can render readily available. We want those who complain that life is dull, or dreary, or a failure, to wake up and resolve to help themselves by helping others with personal service through our Bureau. We want those who are awake already, and many active workers, to lend us their sympathy and give us a hand now and then in the districts where they spend their lives. We want to help them to help those to whom they at present minister, where they find that local facilities are lacking, or other matters cannot be met without going outside for advice or help.

### Give us your Best only

We want those who need help, and those who are willing to give help or to take charge of cases, with or without payment, to recognise that this is an earnest attempt to do a great good, and to be honest themselves and perfectly frank in all their communications with us, as well as in the claims advanced in regard to the accommodation offered or their own qualifications. *One thing we cannot do under any circumstances, and that is to prescribe.* It is altogether foreign to our purpose, and we say this now because we are getting letters asking for this kind of assistance, which is outside our province and purpose altogether.

### Revealed Difficulties already.

We have a letter this week from someone who gives a name which looks like "Hospital" with a name in front of it, which is borne by a medical man, when there is no medical man of that name at the place mentioned. The writer then refers to her country house not far from London, where, we presume, the medical man in question resides. Finally, she offers a home for an invalid child, without stating whether she wishes to make a business arrangement or desires to take the child free, as a good Samaritan. "Sunshine" must excuse us for pointing out that this kind of letter is not a help, but a hindrance both to the writer and the editor. Every fact that is material is omitted from it, and so the writer's and our time is wasted. Yet time is money, and in regard to this Bureau time may mean life or death to someone, for help must always be useless when it comes too late.

### Material Facts.

What do we mean by material facts? That depends upon the object each correspondent has in addressing the editor. Let us give a few examples in order.

*Accredited Correspondents.*—If willing to co-operate, offer your services, send evidence that is reliable of your experience and qualifications, state plainly your position and willingness to help by sending a report on (*a*) cases, or (*b*) homes, houses and apartments where cases are received on payment, or (*c*) of the circumstances and requirements of a case resident in the district where you reside.

*Helpers who offer Personal Experience.*—State your experience and what you think you can do in the way of looking after a particular case or cases in your district, or by making the Bureau known and getting the people who have to do with these cases, who work amongst the poor or are connected with institutions, or in some way are interested—the clergy, ministers of religion, school teachers, district nurses and others—to use the Bureau and take an interest in it.

*Inquirers.*—State exactly what you require, the nature, character, and circumstances of the case you are interested in, the kind of accommodation desired, and if any and what payment can be made.

*Offers of Accommodation.*—First apply to be registered on our list of approved accommodation. To secure this you must give two testimonials from responsible people, stating the nature of the accommodation offered, the kind of case you have had experience in, and the payment, if any, required. All such correspondents, when approved, will have the privilege of advertising in this section of the paper as and when vacancies may occur. All letters to be addressed to "Bureau," care of the Editor, The Hospital, 29 Southampton Street, Strand, London, W.C.

### Wake Up, Everybody!

We want prompt action on the part of our readers in the matter of this Bureau, backed by the best side of their character and the best brains which they may possess.

*Note.*—We hold over several cases and other matters till next week, because the first thing is to organise and form our Brotherhood of Personal Service. (*N.B.*—Our Brotherhood will include both sexes.)

### How to Make the Bureau a Success.

Everyone who uses or wishes to help our Bureau of Information must be kind enough to observe the following rules:

(1) Postal replies cannot be given. Exception will only be made in very special cases at the Editor's discretion.

(2) Queries or answers relating to different cases must be written, or preferably typed, on separate sheets of paper, and the writing must never be crossed.

(3) Questions and replies received not later than Wednesday will, as far as possible, be dealt with in our issue of the following Saturday week.

(4) Letters must have attached the name and address of the sender, with a pseudonym for publication if preferred.

(5) *All communications* for this department *must be accompanied by the coupon* to be found at the bottom of the third page of the cover on the inside, and should be addressed to the Editor of the Bureau of Information, c/o The Hospital, 29 Southampton Street, Strand, London, W.C.

*Notice.*—We invite workers of special experience and knowledge to offer help in replying to questions relating to their special department.

### Information Given.

"Nora."—Please send a coupon. The Holt-Ockley system is now the Cottage Benefit Nursing Association, full particulars of which will be found on page 161 of "How to Become a Nurse" (London: The Scientific Press, Ltd.).

"Fish."—We never prescribe. Have you tried St. George's, Westminster, or St. Thomas' Hospitals, all close at hand?

### A Modern Hospital Dinner.

THE ESSENTIALS TO COMPLETE SUCCESS.

WRITING on this topic two years ago, we maintained that an absence of success where a festival dinner is concerned, even in the present time, means, at the root of things, an absence of sufficient enterprise, devotion and knowledge on the part of those responsible for its organisation. Remarkable evidence in support of this view is afforded by the success of the festival dinner of the City of London Hospital for Diseases of the Chest, Victoria Park. It was a bold step and a generous one for Mr. Andrew Motion to take, to accept the chair for this festival dinner, which was held at the Whitehall Rooms on November 22. Few men have suffered more than Mr. Motion from legislation which had much of its origin in prejudice and venom. He might well have acted on the principle that, when party government robs a man of much of the savings which he has accumulated by hard work and pluck and enterprise during a long and busy life, he is not further called upon to give either his time or his money in the cause of charity. This is not Mr. Andrew Motion's way. His heart is in the right place, and when the needs of the sick invite his sympathy they receive a response so liberal as to be quite exceptional in these days of restricted gifts.

As a speaker Mr. Motion is terse, pointed and successful. He began his speech by reminding those present that they had just enjoyed the jam and now must take the powder. He was careful to honour the names of previous chairmen and friends of the hospital who had done yeoman service for it, and paid a tribute to the Jews, who, under the leadership of Sir Edward Sassoon, were largely represented in the subscription list. He then took the audience in hand and reminded the company of the remarkable fact, which the plan of the tables revealed, that quite half the guests were artisans. He showed that the hospital received £4,000 from the King's Fund, £1,000 from the Sunday Fund, £1,500 from the working men, including Hospital Saturday, and £3,500 from annual subscriptions, making £10,000 towards an expenditure of about £13,000. The guests before him therefore knew that at least £3,000 more was required. That was a large sum and took some courage to work for, when every one of them was vis-à-vis with the methods of the present Government and the increased taxation which fell from them thick as leaves in autumn. Yet the voluntary hospitals, all the world admitted, were the great safeguard for sick people needing hospital care, because the patient as a human being was always the first consideration in a British hospital, in contrast with many State hospitals, where the patient was a mere object for scientific research. He had worked hard for the dinner, or he never would have consented to take the chair. Responsibility means work, and there should never be honour attaching to any post which did not require both. He had pleasure therefore in saying that a little over £3,000 had been contributed, and he appealed to those present to make it up to round figures and so constitute a record collection.

Then the fun began. At the tables were seated representatives of the North London Railway, the Bethnal Green Working Men's Society, the St. James' Philanthropic Society, the Mutual Friendly Aid Society, the Lion Hospital Society, the Good Intent Aid Society, the Excelsior Philanthropic Society, the Albert and Victoria Society, the U. A. O. Druids, the Haggerston Hospital Society, the Hackney Road Society, and the North London Philanthropic Society. The first working-men's representative stood boldly up in response to the chairman's invitation, and said that the men he represented would undertake to increase their contribution by fifty guineas; the next speaker on his mates' behalf promised ten guineas; the next twenty-five pounds; the next pledged himself to increase very largely the donation he brought for the hospital; the fifth guaranteed twenty-one pounds; the sixth said he thought that a hearty vote of thanks was due to the workmen for their gifts; the seventh promised thirty pounds more. Mr. Motion had expressed gratification, and the list of those present proved that he was supported by the presence of every director of his companies, and by most of the staff engaged with him in business. It was therefore with enthusiasm that the eighth speaker declared himself the representative of the clerical staff at the City of London Breweries, who promised to give an additional twenty pounds. So the money was forthcoming, and the £3,250 desired was secured.

One pleasant feature of this dinner was the cordiality exhibited and the grateful recognition extended to the staff of all grades by the Chairman of the Hospital, Mr. Robertson, who dwelt with pleasure on the fact that the Ladies' Association had contributed £2,000 towards a sanatorium which the hospital badly required. He further dwelt upon the valuable services and commendable energy of the Secretary, Mr. George Watts, to whom every credit is due for the excellent arrangements. Dr. T. Glover Lyon, who replied for the medical staff, bore the warmest testimony to the great services which had been rendered to the hospital by the resident medical officer, Mr. W. W. Jameson, M.A., M.B. This hospital is evidently fortunate when the medical staff can record that the principal medical officer has brought order and discipline and the best of good feeling amongst all classes of residents, *i.e.* patients and staff. Again, the general testimony is that the Secretary's (Mr. George Watts) great merit is that he unites the Board and has the knack of making everybody feel welcome and appreciated, so that the work progresses with commendable smoothness and the maximum of success. We noted the alertness of Mr. George Watts, for directly the £3,250 was pouched, he began to work up an interest in favour of special gifts towards a sum of £1,550 required to effect improvements in the heating arrangements, the renewal of the chimney-stacks and stonework, and the re-flooring of the wards and recreation-rooms.

